# Intraperitoneal Oxygen/Ozone Treatment Decreases the Formation of Experimental Postsurgical Peritoneal Adhesions and the Levels/Activity of the Local Ubiquitin-Proteasome System

**DOI:** 10.1155/2011/606718

**Published:** 2011-09-29

**Authors:** Clara Di Filippo, Annalisa Capuano, Barbara Rinaldi, Margherita Luongo, Biagio Lettieri, Francesco Rossi, Michele D'Amico

**Affiliations:** ^1^Department of Experimental Medicine, Section of Pharmacology “L. Donatelli”, 2nd University of Naples, 80138 Naples, Italy; ^2^Department of Anesthesiological, Surgical, and Emergency Sciences, 2nd University of Naples, 80138 Naples, Italy

## Abstract

We have investigated whether an oxygen/ozone (95%O_2_/5%O_3_) mixture would have potential against the formation of experimental postsurgical peritoneal adhesions. In two groups of rats, one control intraperitoneally injected with 3 mL/rat of O_2_ and one intraperitoneally injected with oxygen/ozone mixture (3 mL/rat equivalent to 300 **μ**g/kg ozone), we induced a midline laparotomy and an enterotomy at the level of the ileum to encourage the formation of peritoneal adhesions. Samples were taken from the parietal peritoneal tissue to assess the formation of adhesions 0 and 10 days after the surgical procedure and to assess the levels of ubiquitin and 20S proteasome. We found decreased formation of postsurgical peritoneal adhesions after treatment of the rats with 300 **μ**g/kg ozone associated with a decreased levels of ubiquitin and 20S proteasome subunit within the adhered tissue. Oxygen/ozone mixture is potentially useful for approaching the post-surgical peritoneal adhesions, and the UPS system is involved in this.

## 1. Introduction

Postoperative adhesions by occurring in approximately 90% of patients undergoing abdominal surgery determine postoperative morbidity and mortality [1, 2]. They are connective bridges between adjacent portions of the peritoneum [1] and develop following an extensive inflammatory process within the tissue that involves mediators of inflammation or not.

Adhesions recognize their genesis in mesothelial cells, stimulated by the trauma surgery, which promote the conversion of fibrinogen into fibrin, releasing thromboplastin. Fibroblasts lay collagen fibrils whereas there is fibrin, and so becoming adhesions [3]. Due to the putative molecular mechanisms underlying the postsurgical peritoneal adhesions, various strategies have been proposed to solve the problem. These passed from the use of laparoscopic surgery, pharmacological prevention with drugs acting on fibrinolysis, interferons, and medical anaesthetic gases [4–10]. In addition an important component of the adhesion formation is the inflammatory response occurring within the peritoneum [4, 11, 12].

However, a nonspecific approach using an oxygen/ozone gas mixture might lead to clinical benefit in patients with postsurgical peritoneal adhesions. Such an approach seeks to attenuate the inflammatory response [13] in the unfolding process of adhesions. In this context, our study evaluated whether the application of a medical gaseous mixture of oxygen/ozone during the surgery can be suggestive of a reduction of the inflammatory events that in cascade lead the postsurgical peritoneal adhesions formation. 

## 2. Methods

### 2.1. Surgical Procedure

All experimental procedures and protocols used in this study were approved by the Animal Care Ethical Committee of the 2nd University of Naples. 

The surgery was performed as described by Di Filippo et al. [4]. Male Sprague-Dawley rats (*n* = 15) were marked with a pencil as 1 to 15, anesthetized with urethane (1.2 g/kg ip), subjected to midline laparotomy. A sample of parietal peritoneal tissue was taken, and an enterotomy was performed at the level of the ileum. The surgical incision was sutured with absorbable surgical wire 4/0 in order to induce an inflammatory peritoneal insult. All of the surgical procedure was then ended by a nonabsorbable suture, and the rats were placed in the recovering room for awakening. Ten days after the surgery (time T1) the rats were subjected to another laparotomy having particular attention to keep always the same rat numbering, had a new tissue sample taken, and were assayed for the development of peritoneal adhesions by means of qualitative and quantitative evaluation. A score from 1 to 6 was established, and it was given as follows: 1 to the presence of poor and lapse adhesions in a limited peritoneal zone, 2 to the presence of poor adhesions in an extended zone, 3 to the presence of several lapse adhesions into the peritoneum, 4 to the presence of localized dense adhesions, 5 to the presence of extended dense adhesions with access to peritoneal cavity, and 6 to the presence of extended dense adhesions and impossible access to peritoneal cavity. The surgery and the scoring were always done by the same person. Biopsies of peritoneal tissue were snap frozen and used to determine levels and activity of the UPS system. 

#### 2.1.1. Experimental Groups

The study was conducted on male Sprague-Dawley rats (4–6 months old and weighing 250 g) divided into the following experimental groups: group A (*n* = 15) induced with the surgical procedure + oxygen (100% O_2_, 3 mL/rat, injected in the abdominal cavity) and group B (*n* = 15) induced with surgical procedure + oxygen/ozone mixture (95%O_2_/5%O_3_, 3 mL/rat equivalent to 300 *μ*g/kg ozone injected in the abdominal cavity). Both of the groups were first treated with half volume 1 hour before surgery; a second injection with the other half volume was given 1 hour after the surgical procedure (total volume and doses as specified). There were no significant side effects after injections in both A and B groups.

#### 2.1.2. Biochemical Parameters Assessed

The frozen tissue samples were homogenized in 50 mM Tris-HCl (pH 7.2) containing leptin 1 *μ*M, pepstatin A 1 *μ*M, and phenyl methyl sulfonyl fluoride 200 *μ*M and centrifuged for 10 min at 10,000 ×g at 4°C.

200 *μ*L of homogenate was used to determine total protein according to the Bradford's method. The levels of ubiquitin were quantified by commercial ELISA kit (Wuhan EIAab Science Co., Wuhan, China) For the quantitative measurement of the 20S proteasome, a specific SDS activation kit (Boston Biochem, USA) was used following the instructions of the manufacturer.

#### 2.1.3. Statistics

Data are presented as mean ± S.E. Continuous variables were compared among the groups of rats with one-way analysis of variance (ANOVA) for normally distributed data and the Kruskal-Wallis test for nonnormally distributed data. When differences were found among the groups, the Bonferroni correction was used to make pairwise comparisons. A *P* < 0.05 was considered statistically significant. All calculations were performed using the SPSS2 software.

## 3. Results

Starting from day 10 after surgery, the rats treated with the 3 mL/rat 100% oxygen showed a high score for the development of peritoneal adhesions (data not shown). After 10 days from surgery, 7 of the 15 rats treated with oxygen 3 mL had the highest score assigned for the presence of peritoneal adhesions. They showed presence of dense adhesion and impossible access to peritoneal cavity with a score of 6 assigned to them (Table 1). Three rats were assigned to a score of 5 with a presence of adhesion disseminated within the peritoneum but still keeping the peritoneal access (Table 1). The other rats showed overall lapse adhesions in limited peritoneal zones and gained a score from 1 to 4 (Table 1).

**Figure 1:**
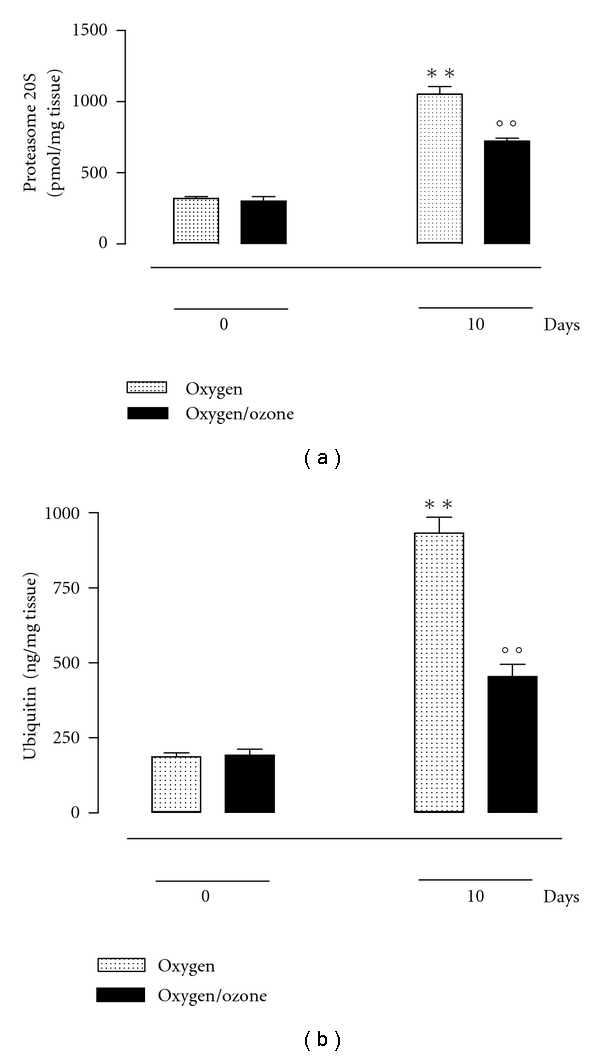
Levels of proteasome 20S (a) and ubiquitin (b) in the peritoneal tissues from rats during peritoneal adhesions development after time 0 and 10 days in the group treated with oxygen (vehicle control) and oxygen/ozone mixture (3 mL, equivalent to 300 *μ*g/kg). The differences from time 0 are considered with *P < 0.01, and the differences from the group treated with oxygen are shown as °*P* < 0.05  and °°*P* < 0.01.

In parallel with the development of peritoneal adhesions, there was an increase in the levels of ubiquitin and the 20S proteasome within the adhered tissue. For both of the markers, this increase was significant (*P* < 0.01 versus time T0) 10 days after surgery (time T1) (Figures 1(a) and 1(b)).

Treatment of rats with 300 *μ*g/kg oxygen/ozone mixture prior to the induction of the surgical peritoneal insult resulted in reduced score for formation of postsurgical peritoneal adhesions and reduced levels of ubiquitin and 20S proteasome within the adhered tissue. The evaluation of the peritoneal adhesions score, as shown in the Table 1, indicates a substantial decrease of adhesions in the group of rats treated with 300 *μ*g/kg oxygen/ozone mixture. This was accompanied by a significant reduction of the levels of both ubiquitin and proteasome 20S (*P* < 0.01 versus time T0) in the peritoneal tissue 10 days after surgery (Figures 1(a) and 1(b)).

## 4. Discussion

Until few years ago to prevent the formation of adhesions, the focus was mainly posed on the procedures to be implemented during the surgery. It is worth mentioning in this regard, as there is considerable consensus on the laparoscopic surgery as a procedure associated with less development of adhesions compared to open surgery in the international arena [4, 5]. However, in recent years, numerous studies have been aimed at a likely pharmacological prevention of postsurgical adhesion formation as, for example, some authors proposed the use of statins as they are considered able to increase the peritoneal fibrinolysis [8]. Other authors have suggested that interferon-gamma is a possible therapeutic target to prevent the formation of adhesions, due to its crucial role in the differential regulation of PAI-1 and t-PA, which are involved in this process [9]. However, other ones suggest the use of antitack agents such as carboxymethylcellulose and hyaluronic acid resorbable membranes. Finally, it is also alleged the possible use of propofol [10]. 

Here, we show that a mixture of oxygen/ozone applied to rats prior to the induction of peritoneal injury exerts a protective action against the formation of postsurgical peritoneal adhesions. 

Experimental and clinical evidences have proved advantageous effects of oxygen/ozone therapy in several pathologies characterized by a cellular oxidative and inflammatory burden, including renal injury, cardiopathy, atherosclerosis and septic shock [14–19]. Recent data imply that ozone is located in atherosclerotic arteries, and it has been proposed that it may be important in additional human inflammatory diseases [20]. Not last, oxygen/ozone administration has been shown beneficial in the prevention/reduction of the myocardial tissue damage which follows an ischemic event [21]. It protects the heart from acute myocardial infarction through a series of local events that include, among the others, increase of eNOS activity and endothelial progenitor cells recruitment [22]. Therapeutic efficacy against wound healing and limb salvage in patients with critical limb ischemia are also due to oxygen/ozone [23].

In a previous research, Di Filippo et al. [4] found that postsurgical peritoneal adhesions occur in rats strictly related to the time from the surgery and have severity depending on the inflammatory response occurring within the peritoneal specimens, which deteriors the peritoneal matrix. During this phase, leukocytes, cytokines, chemokines, and cell adhesion molecules alters the initial tissutal equilibrium predisposing it to the formation of adhesions [4]. In the present study, we show an involvement of the ubiquitin-proteasome system (UPS) in the tissutal alterations that follows a surgical intervention and development of peritoneal adhesions. This latter relates to the severity of the adhesion; animals that show high levels of UPS activity also have severe adhesions, whilst animals that show low levels of UPS have moderate adhesions. 

UPS, generally known as the major pathway for nonlysosomal intracellular protein degradation in eukaryotic cells, was discovered in the eighties by the pioneering work of Goldberg, Hershko, and their collaborators, using reticulocyte lysates [24–26]. The UPS usually degrade proteins in two steps. First, the substrate is covalently modified by addition of a polyubiquitin chain, through an enzymatic cascade that involves three classes of factors: E1, the ubiquitin- (Ub-) activating enzyme [27], E2, a member of the family of Ub carriers [28], and E3, a member of the very large family (several hundred members) of the so-called Ub ligases [29], which specifically recognizes and recruits the substrate of the ubiquitylation reaction. Second, the ubiquitylated protein is usually addressed to and degraded by the 26S proteasome, a giant multisubunit and multicatalytic protease [30]. Due to its multiple roles, the proteasome is essential in eukaryotes, and its dysfunction has deleterious effects for the cell or the organism as a whole. In humans, UPS deregulation has been implicated in a number of pathologies such as cancer, autoimmune diseases, neurodegenerative diseases, or viral infections. As a consequence, the proteasome is seen as a potential therapeutic target in many pathologies [31], including inflammation [32] and now tissue degeneration. 

The present study demonstrates that the activity of the UPS system, measured on adhesions biopsies, is modulated by the treatment with the oxygen/ozone mixture. Following oxygen/ozone treatment, the expression and the activity of this system is diminished. An intriguing and somewhat imaginative scenario would suggest that oxygen/ozone may be able to inhibit the UPS activity in adhered tissue, possibly leading to reduced local inflammatory boost and adhesions formation.

In conclusion, the absolute novelty of this study is that an oxygen/ozone mixture reduces the peritoneal adhesions associated with a reduction of the ubiquitin-proteasome system activity within the adhered tissue.

## Figures and Tables

**Table 1:** Score assigned to rats 10 days after the induction of the procedure for the development of peritoneal postsurgical adhesions: 1, presence of few loose adhesions in a limited area of the peritoneum. 6, presence of dense adhesions and failure in access to the peritoneal cavity. The rats (*n* = 15 for each group) were treated with oxygen (vehicle control, 3 mL) and with oxygen/ozone mixture (3 mL, equivalent to 300 *μ*g/kg). The symbol + indicates the animals showing adhesion.

Adhesions score	Oxygen (3 mL)	Oxygen/ozone (3 mL; 300 *μ*g/kg)
1	+	++
2	++	+++
3	+	++
4	+	++
5	+++	++
6	+++++++	++++
